# Year Book of American Contributions to Medical Science and Literature

**Published:** 1860-12

**Authors:** O. C. Gibbs

**Affiliations:** Frewsburg, Chautauque County, N. Y.


*Circular. The Undersigned proposes to issue a yearly volume with the following title: "Year Book of American Contributions to Medical Science and Literature."*

It is designed that Part *First* of each volume, shall comprise an arranged and classified *Summary* of, and index to, all the important original papers found in the various Medical Journals of this country, for the year immediately preceding. Part *Second* will comprise a *Summary* of, and index to, all papers found in the published transactions of the National and the various State Medical Societies. Part

*Third* will embrace *Reviews* of all medical books of American authorship, published during the year, with a *Summary* of all the novelties in opion or practice therein. A copious index will complete the work. To the above plan and arrangement, such other additions shall be made as time and circumstances may suggest. The first volume will be issued early in the year 1861.

For the preparation of our *Monthly Summary of American Medical Journalism*, for the *A. M. Monthly*, we have arranged for all the American Medical Journals (over thirty in number) and at least four of the best European. To facilitate our design, we request a *continuance* of the *exchange*.

American authors of medical works, and publishers of the same, are requested to send to our address a copy of their respective works and publications. Medical Societies that publish their transactions, will, we trust, be kind enough to send a copy of the same to our address. For this kind of an exchange from Journal publishers, authors, book publishers, and Presidents of Societies, we shall be happy to reciprocate by an *exchange of publications*.

The importance of a work of the characters as above, which shall comprise *all there is of interest* in the more than 30 Journals, medical books issued for the year, and the various society transactions, will be readily conceded by all.—We can not prepare the work and publish it at a pecuniary loss, and hence the object of this Circular is to request that all physicians who would encourage the work, and become subscribers to the same, *would send us their names* AT ONCE—payment to be made only on the publication of the work.—The work shall contain from 500 to 800 pages, to be substantially bound, and furnished at the very low price of *Three Dollars*. That we may know whether the work is to receive sufficient encouragement to justify its completion and publication, *we request that subscribers' names* MAY BE SENT IN IMMEDIATELY. As a special favor we request that our friends will not allow a day to pass before responding to this Circular.

If Journal publishers will take sufficient interest in this National Enterprise, to publish our Circular, we shall be happy to remunerate, should the enterprise more than pay expenses.

All books, Journals, Published Transactions, and Names of Subscribers, should be directed to

O. C. GIBBS, M. D.,
Frewsburg, Chautauque County, N. Y.

